# Malnutrition and Obesity in Patients with COPD Exacerbation, Insights from the National Inpatient Sample

**DOI:** 10.2174/0118743064322829240801094830

**Published:** 2024-08-23

**Authors:** Mohamad El Labban, Roba El-Zibaoui, Syed Muhammad Usama, Fayreal Niaz, Abbe Cohen, Peter Krastev, Syed Khan, Salim Surani

**Affiliations:** 1 Department of Medicine, Mayo Clinic College of Medicine and Science, Rochester, MN, USA; 2 School of Medicine, American University of Beirut, Beirut, Lebanon; 3 Department of Internal Medicine, Nazareth Hospital-Trinity Health Mid Atlantic, PA, USA; 4 School of Medicine, St. George’s University, Grenada, West Indies; 5 School of Medicine, Kansas City University College of Osteopathic Medicine, Kansas City, MO, USA; 6 Department of Medicine & Pharmacology, Texas A&M University, College Station, TX, USA

**Keywords:** COPD, COPD exacerbation, Malnutrition, Obesity, Sarcopenic obesity, Mechanical ventilation, Nationwide study

## Abstract

**Background:**

The obesity paradox suggests that obese patients with Chronic Obstructive Pulmonary Disease Exacerbation (COPDE) may have better outcomes. COPD patients are at a higher risk of becoming malnourished, which has been linked to poor outcomes.

**Objective:**

This paper aims to study the impact of malnutrition in patients with and without obesity hospitalized with COPDE.

**Methods:**

Our retrospective study analyzed data from the National Inpatient Sample dataset between 2017 and 2020 to observe patients who were hospitalized with COPDE. The patients were divided into two groups: with and without malnutrition. The outcomes included all-cause mortality, invasive mechanical ventilation, length of stay, and total charge. We adjusted for confounders using multivariate regression model analysis.

**Results:**

The study involved 392,920 patients with COPDE, out of which 5720 (1.45%) were diagnosed with malnutrition. Most of the patients in both groups were female, white, and under Medicare coverage. The mean age was higher in patients with malnutrition (67.6 *vs*. 64 years). In both groups, the rates of admissions were lowest in 2020 compared to three years prior. The rates and adjusted odds ratios of all-cause mortality were higher in patients with malnutrition (3.59% *vs*. 0.61%, P <0.01; adjusted odds ratio (aOR) 2.36, P<0.01, CI 1.8-3.7). We observed comparable findings when using invasive mechanical ventilation (13.2% *vs*. 2.82%, P<0.01, aOR 4.9, P<0.01, 3.9-6). Malnourished patients had a lengthier hospital stay and a greater total charge.

**Conclusion:**

Malnutrition was identified as an independent risk factor associated with worse outcomes in obese patients admitted with COPD exacerbation.

## 1 INTRODUCTION

Chronic obstructive pulmonary disease (COPD) is an obstructive lung disease that stems from harmful substances that cause progressive airflow limitations and destruction of lung tissues [1]. COPD exacerbations (COPDE) are transient episodes of worsening respiratory symptoms and, if increased in frequency, may lead to detrimental disease progression with an increased risk of death [2]. COPDE outcomes vary based on a multitude of different factors, including demographic factors such as age, comorbidities, and baseline pulmonary function. Many studies have identified a correlation between poor clinical outcomes and elderly patients, as they are more likely to naturally decline lung function [3]. Good prognostic factors include vaccination, pulmonary rehabilitation, and smoking cessation, which enhance patient outcomes and decrease exacerbation frequency [4]. A problem that has been overlooked is how malnourishment can affect patients with COPD. According to the World Health Organization, malnutrition refers to deficiencies or excesses in nutrient intake, imbalance of essential nutrients, or impaired nutrient utilization [5]. Malnourishment causes respiratory muscle wasting and weakness, leading to a decline in respiratory effort [6]. Patients with COPD who are also malnourished have a greater decline in respiratory muscles, which in turn leads to increased severity of the disease [7]. Sarcopenia is another factor that can play a part in this study. Sarcopenia is an age-related loss of muscle mass and muscle strength, and when associated with chronic illnesses such as COPD, it can cause further deterioration in muscle mass [8]. The obesity paradox challenges the normal stigma behind obesity and the link to increased mortality [9]. The obesity paradox was first discussed in the early 2000s and has continued to be a topic of interest for various diseases [10]. In the past, the management of obesity primarily entailed lifestyle modifications like diet and exercise. However, there has been increased attention on certain drugs that aid in weight loss, such as glucagon-like peptide one receptor (GLP-1) agonists [11]. Healthcare providers are increasingly turning to these medications to better control the obesity crisis, which in turn may improve cardiovascular outcomes but are worsening respiratory outcomes in patients with COPD [12]. Malnutrition can be diagnosed using the criteria outlined in Table **1**. For instance, if a patient with a BMI of 40 (which is considered obese) experiences a COPD exacerbation and is assigned an ICD-10 code E43 by the dietician, the patient may still be classified as obese while meeting the criteria specified in Table **1**. This is because the criteria involve a percentage change from the baseline. Therefore, a >2% weight change in a week, along with reduced energy intake, would fulfill the criteria for malnutrition despite still being classified as obese based on the BMI. The “Obesity Paradox,” is mentioned in the discussion. This study aims to explore if obese patients with malnutrition who are admitted for COPDE have a different outcome compared to their counterparts without malnutrition.

## 2 MATERIALS AND METHODS

### 2.1 Design and Description of the Database

We used the National Inpatient Sample (NIS) from 2017 to 2020, which is the largest inpatient hospital discharge database in the US [13]. The database approximates a 20% stratified sample of discharges from US hospitals, excluding rehabilitation and long-term acute care hospitals. Cost information was obtained from hospital accounting reports in the Healthcare Cost Report Information System (HCRIS) files collected by the Centers for Medicare & Medicaid Services (CMS).

### 2.2 Selection of Cases and Outcome Variables Examined

The NIS is a publicly available large dataset managed by the Healthcare Cost and Utilization Project and the Agency for Healthcare Research and Quality. The database approximates a 20% stratified sample of discharges from US hospitals, excluding rehabilitation and long-term acute care hospitals.

The selection of cases and outcome variables section is based on ICD-10 coding. Codes reflecting malnutrition are further elaborated *via* Table **1**. In the NIS dataset, the principal diagnosis is the main ICD-10 (International Classification of Disease, 10th edition, clinical modification [ICD-10-CM]) code linked to inpatient status at admission. Final ICD-10 codes are based on final diagnoses after the hospitalization is complete. The secondary diagnosis refers to the patient's medical condition that is listed after the primary or main diagnosis on the problem list. All procedure codes detected *via* NIS are linked to the admission. Our study identified COPDE as the primary diagnosis. Our inclusion criteria included adult patients (age 18 years or older) with obesity hospitalized emergently with a principal diagnosis of COPDE in the years 2017 to 2020. We then split the patients into two groups: with and without a diagnosis of malnutrition. Patients with malnutrition were identified *via* the ICD-10 codes corresponding to the diagnostic criteria, as shown in Table **1**. Table **S1** included secondary diagnoses that were identified using ICD-10 codes. Within our research, we examined individuals with sarcopenia, a condition characterized by muscle wasting, and accounted for this diagnosis as a potential confounding variable in our regression analysis. This was done to highlight our main focus, which was on the caloric components of malnutrition, specifically how weight loss and decreased body fat could affect individuals. The Charlson Comorbidity Index was used to describe the patients' co-existing medical conditions. From the NIS dataset, outcomes were generated, which included all-cause mortality, in-patient invasive mechanical ventilation (IMV), length of stay, and total charges.

### 2.3 Statistical Analysis

Statistical analyses were conducted using STATA BE Version 17.0. All statistical tests were two-sided, and a p-value of <0.05 was considered to be statistically significant. Chi-square analysis was used to explore the differences in patient characteristics and secondary diagnoses between those with and without malnutrition. We conducted two analyses to assess the results. In the first analysis, we compared the outcome rates of patients with and without malnutrition using chi-square analysis. Additionally, we compared the outcome rates among three groups: group I without obesity and malnutrition, group II with obesity but without malnutrition, and group III with both obesity and malnutrition, using Pearson's χ2 test analysis. This was done to investigate the potential protective effect of obesity on outcomes of acute pulmonary disorders, which is known as the obesity paradox. The isolated impact of malnutrition on outcomes was described using multivariable regression models. In the model, patients with COPDex, without obesity or malnutrition, served as the reference group. The following variables were included in the regression model: year of hospitalization, age, female gender, race (White (as the reference), Black, Hispanic, Asian/Pacific, Native American, other), Charlson Comorbidity Index as categories (Group 1 (Score of one, this was the reference), group 2 (Score of 2), group 3 (score of ≥3), insurance status (Medicare (reference), Medicaid, private insurance, self-pay), Diabetes mellitus II, hypertension, chronic kidney disease, sepsis, dementia, coronary artery disease, chronic heart failure, pulmonary hypertension, and sarcopenia. All regression models in our study had a significant F-value (Prob > F <0.01), indicating that the independent variables can reliably predict the studied variable.

### 2.4 Data User Agreement

The first author, Dr. El-Labban, has successfully completed the data user agreement with HCUP-AHRQ. The HCUP datasets are exempt from institutional review boards (IRB) review as they are publicly available (Federal Regulations 45 CFR 46.101 (b)).

## 3 RESULTS

Between 2017 and 2020, the NIS dataset generated 392,920 unique discharges for obese patients hospitalized with a principal diagnosis of COPDEx. Of the 392,920 adult patients, 5720 (1.46%) were also malnourished, and the diagnosis of malnourishment followed the ICD-10 diagnostic criteria (Table **1**). Our data support this paradox by showing that obese patients with COPD had reduced use of mechanical ventilation, length of stay, and in-hospital mortality even after adjusting for confounders such as sex, race, age, and comorbidities.

Two groups of patients were analyzed based on the presence or absence of malnutrition. Table **2** displays the demographic and clinical characteristics. Both groups had a comparable rate of being female and white. The non-malnourished group had a mean age of 64, while the malnourished group had an average of 67.6 years old. The malnutrition group had a significantly higher percentage of patients with a score of 3 or more on the Charlson Comorbidity Index (64% *vs*. 53%; p-value<0.01) and under Medicare coverage. Clinically significant comorbidities in the malnourished group included dementia, sepsis, chronic kidney disease, and sarcopenia (Table **2**).

Primary outcomes show that the patients who were both obese and malnourished had a significantly higher mortality rate than the group that was solely obese (3.59% *vs* 0.61%; p-value <0.01). The percentage of patients needing invasive mechanical ventilation (IMV), a secondary outcome, was also higher in the malnutrition group, with 13.2% compared to 2.82% in the nonmalnourished group (p-value <0.01). Similarly, the length of hospital stay (7.3 days *vs* 4.4 days) and total charge ($78,483 *vs* $40,975) were both higher in the obesity with malnutrition group (Table **3**).

When compared with control patients who were neither obese nor malnourished, the malnutrition group had a significantly higher mortality rate (3.59% *vs* 0.98%; p-value<0.01) (Table **4**). Patients with obesity but without malnutrition had the lowest mortality rate, even lower than the control (0.61% *vs* 0.98%; p-value<0.01). This trend did not continue with secondary outcomes. Control patients had the lowest chance of needing IMV (2.17%), followed by the non-malnourished group (2.82%), and as seen earlier, the malnutrition group had the highest rate of IMV at 13.2%. Similarly, LOS and total charge were lowest in the control group (3.9 days/ $34909), closely followed by the non-malnourished group (4.4 days/ $40975) and the obesity with the malnutrition group having the longest and most expensive stay (7.3/ $78483).

The obesity without malnutrition group had significantly lower odds of mortality than the control (aOR 0.76; p-value<0.01). On the other hand, individuals with obesity and malnutrition had significantly increased odds of mortality (aOR 2.63; p-value <0.01, CI 1.8-3.7). For the use of IMV, compared to the control group, individuals with obesity without malnutrition have increased odds of needing IMV, and individuals with both obesity and malnutrition have even greater odds of needing IMV (Table **5**).

On average, the obesity with malnutrition group stayed 0.45 days longer than the control group (Table **6**). Similarly, individuals with obesity and malnutrition also had a significantly longer length of stay. On average, the obesity without malnutrition group had a significantly higher charge than the control and the malnutrition group had an even higher average charge than the control at $38,413 (Table **6**).

## 4 DISCUSSION

The world is amid an unprecedented obesity epidemic, a complex disorder characterized by poor dietary intake and physical inactivity that alter the body’s microbiome, immunity, and cellular function [14]. This epidemic has not only joined the ranks of chronic diseases but has also superseded malnutrition and infection as one of the leading causes of mortality among the general public [15]. Currently, obesity is classified using the statistical index tool body mass index (BMI), which is based on adults' anthropometric height/weight characteristics. The BMI classifications and cutoffs have been set by the National Institute of Health (NIH) and the World Health Organization (WHO) [16]. Although BMI does not directly quantify body fat, it correlates with other ways of measuring it. It has been identified as one of the top five risk factors in deaths and disability [16]. Physicians have linked obesity to multiple pulmonary disorders, including obstructive sleep apnea (OSA), asthma, and COPD. The underlying chronic low-grade systemic inflammation accompanying obesity has been associated with pulmonary structure and function changes. Yet, evidence about these changes' mechanisms remains obscure and warrants further investigation [17, 18]. This is because, ironically, an inverse relationship has been observed between BMI and mortality in patients with chronic diseases such as cardiovascular disease, heart failure, type 2 diabetes mellitus, and chronic kidney disease [19, 20]. This phenomenon, described as “the obesity paradox,” is often displayed as a reverse “J-shaped” curve with improved survival among overweight and obese patients when compared to normal-weight individuals [21]. This is in sharp contrast to the “U-shaped” graph in the general population, where an increase in mortality was noted at both extremes of BMI [21]. Consistent with these premises, our retrospective study of patients with obesity hospitalized emergently with COPD provides support for the obesity paradox. In our study sample, obese patients with COPD had reduced use of mechanical ventilation, length of stay, and in-hospital mortality even after adjusting for confounders such as sex, race, age, and comorbidities.

The prevalence of obesity among COPD patients in the United States is approximately 65%, with more than half of the subjects having a BMI measurement ≥30 kg/m2 [22]. Despite the widespread prevalence of obesity in this population, its impact on clinical outcomes remains inconsistent [21]. Numerous authors have stated that the obesity paradox is grossly overemphasized in importance and may be the result of various biases, such as socioeconomic status, smoking, or medical care, among overweight patients [23]. This notion has been supported by the findings of the COPDGene study, which reported that the combination of obesity and COPD is associated with poor clinical outcomes, increased use of noninvasive and invasive ventilation, and longer lengths of hospital stay [24, 25]. This was assumed to be due to the altered diaphragmatic movements, which potentiate the pre-existing ventilation/perfusion mismatch, micro atelectasis, airway closure, exercise intolerance, dyspnea, increased risk of exacerbations, and poor quality of life [26]. Moreover, as emphysema is independently associated with both weight loss and high rates of mortality, some authors have speculated that the “proposed” protective effect of obesity against mortality may, in actuality, be related to reduced emphysema severity rather than weight per se [26]. In other words, this “paradox” represents a case of reverse causation as weight loss is often correlated with increasing severity of illness [27]. Similarly, other studies also have stated that since the forced expiratory volume in the first second (FEV1) and forced vital capacity (FVC) both decrease with increasing BMI, obese subjects with low FEV1 may be wrongly classified as having a worse bronchial obstruction when compared to their real clinical stage due to failure to consider the underlying restrictive effect of obesity [26].

Nevertheless, other trials have shown that the increased mortality rates among underweight or malnourished patients continue to exist despite adjusting for possible confounders, including FEV1, suggesting that disease severity alone does not explain the survival advantage observed among obese patients [28]. Likewise, the BODE composite score, which is frequently used to predict the prognosis of COPD, has repeatedly shown that BMI values <21 are associated with an increased risk of death in population studies [28, 29]. The impact of obesity on pulmonary physiology has also been explored with evidence to suggest that obesity is associated with preserved elastic recoil, lower dyspnea intensity ratings, and better ventilation when compared with FEV1-matched normal-weight patients with COPD [18]. Surprisingly, some studies have reported that a combination of obesity and COPD may not be associated with diminished exercise capacity or dyspnea despite previous assumptions [30]. These studies have shown that obesity may ironically be a protective factor as patients demonstrated less lung hyperinflation and had a larger inspiratory capacity/total lung capacity ratio than their lean counterparts [30]. Furthermore, other recent large-scale international trials such as SUMMIT and TORCH have also shown that obese patients with COPD may have a lower frequency of exacerbations in addition to lower rates of pneumonia, hospital readmissions, use of mechanical ventilation, and in-hospital mortality even after matching patients for underlying comorbidities [21]. However, it is important to acknowledge that this trend was not apparent in COPD patients with BMI ≥40 kg/m2 [10]. While some have suggested that the increased mortality rates among morbidly obese patients negate the obesity paradox, others have, in opposition, stated that such patients often die from cardiovascular and metabolic complications of obesity that have time to develop due to longer survival time [31].

As the relationship between obesity and mortality among COPD patients remains controversial, several theories have been proposed to explain the ongoing discrepancies in clinical findings. Some authors have postulated that BMI is a misleading indicator for survival as it fails to consider the body composition or cardiorespiratory fitness of patients [26]. As such, one must differentiate between various phenotypes of obesity as it does not only alter the treatment approach but also contributes to predicted morbidity and mortality of patients. Some phenotypes described include obesity based on BMI, obesity with malnutrition, or sarcopenic obesity [15]. Although BMI and malnutrition have defined criteria, the same does not apply to sarcopenia. As there remains to be no consensus about the definition of sarcopenia, it is often characterized by age-related progressive loss of skeletal mass, function, and weakness with a concomitant increase in fat mass [32]. Such delineations acknowledge that obesity and malnutrition are not mutually exclusive [33]. Therefore, COPD patients may still be classified as malnourished despite being classified as overweight or obese on the BMI scale [15]. Findings from our study further support the role of malnutrition in the overall prognosis of obese COPD patients. Among our sample, patients identified to be malnourished by ICD-10 criteria, regardless of their BMI, were associated with increased length of stay, use of noninvasive and invasive mechanical ventilation, and higher mortality rates.

The underlying association of malnutrition among COPD patients with obesity remains vastly under-recognized and often untreated [34]. Nevertheless, it has been established that the resultant muscle atrophy and a type II fiber shift in malnourished COPD patients are associated with a poor prognosis independent of lung function [35]. Moreover, the greater resting energy expenditure and protein atrophy in COPD patients are often exacerbated by systemic inflammation, hypoxia, and excessive use of systemic corticosteroids [15]. The dysphagia associated with chronic mucus production, mouth breathing, and coughing may further contribute to poor nutritional intake [36]. Consequently, malnourished COPD patients are likely to develop respiratory muscle weakness, leading to impaired gas exchange [25]. This may explain why some studies have found a reduced influence of obesity on mortality after controlling for muscle mass [10]. Similar theories have also been used to explain the association between sarcopenic obesity and COPD [37]. Previously defined as the combination of obesity with age-related muscle loss, sarcopenic obesity is now considered a multifactorial clinical syndrome impacted by physical inactivity, malnutrition, and chronic disease [32]. COPD has been established as a chronic inflammatory condition, both locally and systemically, due to the release of numerous inflammatory mediators [38]. This chronic inflammation mediated by the immune system may be a crucial factor in the development of sarcopenia [39]. With a prevalence of around 15% among COPD patients, sarcopenia is believed to significantly contribute to the catabolic inflammatory processes that lead to impaired lung function and poor health status [40]. Interestingly, several studies have shown that obesity without malnutrition may have a protective effect against sarcopenia due to stronger antigravity muscles and preservation of muscle mass [40]. This finding, however, does not hold for COPD patients with sarcopenic obesity [35]. In such patients, the loss of muscle and the resultant increase in adipose tissue may promote the release of pro-inflammatory adipokines such as resistin, which can lead to a self-perpetuating vicious circle of muscle atrophy due to lipotoxicity, muscle insulin resistance, and mitochondrial dysfunction [32]. Such findings are consistent with the observed results in our population, as patients with sarcopenic obesity were associated with poorer clinical outcomes despite their BMI classification. Therefore, one must be wary of considering malnutrition and sarcopenia, even among obese patients with COPD, as excessive weight loss may adversely exacerbate muscle atrophy and worsen the disease prognosis.

It is important to note, however, that though lean muscle mass has been identified as a positive prognostic factor in patients with COPD, adipose tissue may also offer a protective role [41]. Contrary to what one may assume, a decrease in fat may be an independent risk factor for mortality in COPD patients, regardless of the patient’s baseline fat levels [9]. Possible reasons to explain such results may be the increased energy reserves among obese patients, which may be particularly important during acute exacerbations [9]. Furthermore, adipose tissues have been shown to produce various soluble tumor necrosis factor-alpha receptors, which may attenuate the negative biological effects of TNF-a and other cytokines such as interleukin-6 and interleukin-8 released during periods of inflammation, which may increase the risk of death from cardiovascular disease [42]. Moreover, adiposity has also been found to impair neutrophil signaling and recruitment. In the case of COPD, disease progression and exacerbation are often correlated with increased neutrophil infiltration and degranulation, leading to progressive damage to lung architecture and dysfunctional repair [10]. One may postulate, therefore, that increased adiposity may result in decreased severity and lung damage during exacerbations. Similarly, the increased prevalence of adipokines, such as Leptin, in patients with COPD obesity has been shown to promote the shift of CD4+ T cells to Th1 cells, which may improve macrophage phagocytosis, bacterial clearance and reduce the incidence of bacteremia [10]. Therefore, the improved mortality rates in such patients cannot be solely attributed to protein mass, as the increased adipose tissue may also be associated with better tolerance to inflammation and improved immunity.

Due to the ongoing uncertainty about the ‘obesity paradox’ in patients with COPD, clinicians will continue to face the dilemma of whether to treat obesity and, similarly, how one should approach treatment. Although weight loss may improve cardiovascular outcomes in this population, it may be associated with increased mortality due to the resulting loss of muscle mass, which is detrimental to diaphragmatic and respiratory function [15]. Moreover, multiple studies have found that weight gain or stable weight was associated with better survival among obese subjects with severe COPD when compared to normal-to-underweight subjects [43]. As there are no guidelines about how to manage obesity in patients with COPD, caution is heavily warranted to balance both the beneficial and detrimental effects of weight loss in this population [12]. Despite limited data, there is some compelling evidence to suggest that a combination of GLP agonists, in addition to dietary supplementation and strength-resistance exercises, may yield positive outcomes in this population [34, 44]. This is because the use of GLP agonists, such as Liraglutide and Exenatide, have not only been linked to weight loss but may also have anti-inflammatory properties while also promoting surfactant production and bronchodilation [44]. Nevertheless, trials examining the effects of GLP agonists are scant and often short-lived. Thus, the longer-term effects of such medication remain unexplored. Other proposed weight-loss strategies have focused on using supervised meal replacements and resistance exercise training to reduce BMI without losing lean skeletal muscle mass. Such strategies, however, do not address the underlying inflammation but have been associated with improvements in exercise capacity and BODE scores while mitigating the effect of sarcopenia [34]. Nonetheless, there remains a lack of evidence about how to address obesity among COPD patients, and further research is greatly warranted to develop guidelines that would promote optimal clinical outcomes.

Our study has several limitations. First, the study's retrospective nature limits causal inference and our ability to establish direct causal relationships between malnutrition and outcomes. Secondly, the data we used relied on administrative coding rather than clinical diagnostics such as imaging, which might have introduced biases or misclassifications, even though the NIS offered a large sample size. Our study involved patients who were hospitalized in 2020 during the COVID-19 pandemic. However, underreporting makes it hard to determine the true frequency of infections. We make up for this limitation by presenting data on patients hospitalized before (2017,2018,2019) and after (2020) the pandemic. We factored the year of the admission into the regression model to adjust for that factor.

## CONCLUSION

Although an obesity paradox in COPD has been well-described, it should not be seen as a promotion of obesity in this population, as obesity remains a well-known risk factor for countless diseases. Nonetheless, it is prudent to avoid causal inferences from such observational data, as the obesity paradox may not apply to obese patients with malnutrition or sarcopenia. Given the findings presented above, all COPD patients require ongoing nutrition assessment while recognizing the underlying presence of malnutrition or undernourishment regardless of BMI value. As such, we propose that adopting a multi-disciplinary team approach to allow for ongoing reassessment and nutritional adjustment is an invaluable tool for promoting favorable outcomes in COPD patients.

## Figures and Tables

**Table 1:** ICD-10 malnutrition diagnostic criteria.

**Diagnosis**	**Severe Protein Calorie Malnutrition**			**Malnutrition of Moderate Degree**		
Criteria (at least two must be present)	Acute injury/illness	Chronic illness environmental	Social/behavioral/circumstances	Acute injury/illness	Chronic illness environmental	Social/behavioral/circumstances
Weight loss	>2% x 1 week >5% x 1 month >7.5% x 3 months	>5% x 1 month >7.5% x 3 months >10% x 6 month >20% x12 months	>5% x 1 month >7.5% x 3 months >10% x 6 month >20% x12 months	1-2% x 1 week 5% x 1 month 7.5% x 3 months	5% x 1 month 7.5% x 3 months 10% x 6 months 20% x 12 months	5% x 1 month 7.5% x 3 months 10% x 6 months 20% x 12 months
Energy intake	<50% energy intake compared to estimated energy needs ≥ 5 days	<75% energy intake compared to estimated energy needs ≥ 1 mo	<50% energy intake compared to estimated energy needs ≥ 1 mo	<75% energy intake compared to estimated energy needs ≥ 7 days	<75% energy intake compared to estimated energy needs ≥ 1 mo	<75% energy intake compared to estimated energy needs ≥ 3 mo
Body fat	Moderate depletion	Severe depletion	Severe depletion	Mild depletion	Mild depletion	Mild depletion
Muscle mass	Moderate depletion	Severe depletion	Severe depletion	Mild depletion	Mild depletion	Mild depletion
Fluid accumulation	Moderate to Severe	Severe	Severe	Mild	Mild	Mild
Corresponding ICD-10 Code	E43 Unspecified severe protein-calorie malnutrition	E43 Unspecified severe protein-calorie malnutrition	E43 Unspecified severe protein-calorie malnutrition	E44 Moderate protein-calorie malnutrition	E44 Moderate protein-calorie malnutrition	E44 Moderate protein-calorie malnutrition

**Table 2:** Demographic and clinical patient characteristics by clinical group.

**Characteristics**	**Without Malnutrition**	**With Malnutrition**	**p-value**
**No**	387200	5720	-
**Female, No (%)**	247808 (64)	3735 (65)	0.42
**Age (y)**	64	67.6	-
**Year**	-		<0.01
2017	127776 (33)	2231 (39)	-
2018	100672 (26)	1544 (27)	-
2019	96800 (25)	1201 (21)	-
2020	61952 (16)	744 (13)	-
**Race, No (%)**	-		0.03
White	280178 (72.3)	4176 (73)	-
Black	71090 (18.3)	883 (15.4)	-
Hispanic	24781 (6.4)	423 (7.4)	-
Asian/Pacific	2323 (0.6)	62 (1)	-
Native American	2246 (0.6)	36 (0.6)	-
Other	6582 (1.7)	140 (2.4)	-
**Charlson Comorbidity Index score, No. (%)**	-		<0.01
**1**	77440 (20)	858 (15)	-
**2**	104544 (27)	1201 (21)	-
**>=3**	205216 (53)	3661 (64)	-
**Insurance type, No. (%)**	-		<0.01
Medicare	251680 (65)	4061 (71)	-
Medicaid	75504 (19.5)	1030 (18)	-
Private Insurance	50336 (13)	515 (9)	-
Self-pay	9680 (2.5)	114 (2)	-
**Comorbidities, No. (%)**	-		-
Dementia	9710 (2.5)	275 (4.8)	<0.01
Sepsis	4605 (1.2)	290 (5)	<0.01
DMII	205216 (53)	2875 (50)	0.08
HTN	321376 (83)	4740 (82.7)	0.83
CKD	36784 (9.5)	910 (15.9)	<0.01
Pulmonary Hypertension	27104 (7)	445 (7.7)	0.33
Chronic Heart Failure	69696 (18)	1035 (18)	0.79
CAD	120032 (31)	1835 (32)	0.36
Sarcopenia	16650 (4.3)	335 (5.8)	0.01

**Abbreviations: **DMII: Diabetes Mellitus II; HTN: Hypertension; CKD: Chronic kidney disease; CAD: Coronary artery disease.

**Table 3:** Primary and secondary outcomes.

**Characteristics**	**Obesity without Malnutrition**	**Obesity with Malnutrition**	**p-value**
**All-cause mortality, No (%)**	2362 (0.61)	205 (3.59)	<0.01
**IMV, No (%)**	10919 (2.82)	755 (13.2)	<0.01
**LOS (days)**	4.4	7.3	-
**Total Charge (US $)**	40975	78483	

**Abbreviations: **NIV: Non-invasive ventilation; IMV: Invasive mechanical ventilation; LOS: Length of stay.

**Table 4:** Primary and secondary outcomes (obesity groups *vs*. control).

**Characteristics**	Control*	**Obesity without Malnutrition**	**Obesity with Malnutrition**	**p-value**
**All-cause mortality, No (%)**	18998 (0.98)	2362 (0.61)	205 (3.59)	<0.01
**IMV, No (%)**	42067 (2.17)	10919 (2.82)	755 (13.2)	<0.01
**LOS (days)**	3.9	4.4	7.3	-
**Total Charge (US $)**	34909	40975	78483	

Note: *Absence of obesity & malnutrition.

NIV: Non-invasive ventilation; IMV: Invasive mechanical ventilation; LOS: Length of stay.

**Table 5:** Adjusted* odds ratio (aOR) of all-cause mortality and use of invasive mechanical ventilation.

**Characteristics**	**aOR**	**P-value**	**Confidence Interval**
**All-cause Mortality**			
Controlǂ	Reference		
Obesity without malnutrition	0.76	<0.01	0.69-0.85
Obesity with malnutrition	2.63	<0.01	1.8-3.7
**Use on IMV**			
Controlǂ	Reference		
Obesity without malnutrition	1.13	<0.01	1.07-1.19
Obesity with malnutrition	4.9	<0.01	3.9-6

**Note: **ǂAbsence of obesity and malnutrition.

*variables adjusted for age, female gender, race, Charlson comorbidity index, insurance coverage, sepsis, diabetes mellitus, hypertension, pulmonary hypertension, chronic heart failure, coronary artery disease, chronic kidney disease, sarcopenia.

**Table 6:** Length of stay and total charge adjusted means.

**Characteristics**	Adjusted Means*	**P-value**	**Confidence Interval**
Length of stay			
Controlǂ	Reference		
Obesity without malnutrition	0.45	<0.01	0.41-0.48
Obesity with malnutrition	2.9	<0.01	2.5-3.4
Total Charge			
Controlǂ	Reference		
Obesity without malnutrition	4805	<0.01	4352-5258
Obesity with malnutrition	38413	<0.01	31163-45662

**Note: **ǂAbsence of obesity and malnutrition.

*variables adjusted for age, female gender, race, Charlson comorbidity index, insurance coverage, sepsis, diabetes mellitus, hypertension, pulmonary hypertension, chronic heart failure, coronary artery disease, chronic kidney disease, sarcopenia.

## Data Availability

The data and supportive information are available within the article.

## LIST OF ABBREVIATIONS

COPD: Chronic obstructive pulmonary disease
COPDE: Chronic obstructive pulmonary disease exacerbations
GLP-1: Glucagon-like Peptide One Receptor
NIS: National Inpatient Sample
HCUP: Healthcare Cost and Utilization Project
AHRQ: Agency for Healthcare Research and Quality
ICD-10: International Classification of Disease, 10th edition
IMV: Invasive Mechanical Ventilation
LOS: Length of Stay
BMI: Body Mass Index
OSA: Obstructive Sleep Apnea
FEV1: Fractional Expired Volume in the first second
FVC: Forced Vital Capacity
