# Supplementary material for: Malnutrition and Obesity in Patients with COPD Exacerbation, Insights from the National Inpatient Sample
Source: Open Respir Med J. 2024 Aug 23;18:e18743064322829. doi: 10.2174/0118743064322829240801094830 (PMC11499682; doi:10.2174/0118743064322829240801094830)
Supplement: Supplementary file 1 [file TORMJ-18-e18743064322829_SD1.pdf]

The Open Respiratory Medicine Journal
DOI: 10.2174/0118743064322829240801094830, 2024, 18, e18743064322829
ISSN: 1874-3064


SUPPLEMENTARY MATERIAL

OPEN ACCESS

# Malnutrition and Obesity in Patients with COPD Exacerbation, Insights from the National Inpatient Sample

Mohamad El Labban[1], Roba El-Zibaoui[2], Syed Muhammad Usama[3], Fayreal Niaz[4], Abbe Cohen[4], Peter Krastev[5], Syed Khan[1] and Salim Surani[6,*]

[1]*Department of Medicine, Mayo Clinic College of Medicine and Science, Rochester, MN, USA*
[2]*School of Medicine, American University of Beirut, Beirut, Lebanon*
[3]*Department of Internal Medicine, Nazareth Hospital-Trinity Health Mid Atlantic, PA, USA*
[4]*School of Medicine, St. George's University, Grenada, West Indies*
[5]*School of Medicine, Kansas City University College of Osteopathic Medicine, Kansas City, MO, USA*
[6]*Department of Medicine & Pharmacology, Texas A&M University, College Station, TX, USA*

© 2024 The Author(s). Published by Bentham Open.
This is an open access article distributed under the terms of the Creative Commons Attribution 4.0 International Public License (CC-BY 4.0), a copy of which is available at: https://creativecommons.org/licenses/by/4.0/legalcode. This license permits unrestricted use, distribution, and reproduction in any medium, provided the original author and source are credited.

*Address correspondence to this author at the Department of Pharmacology, Texas A&M University, P.O. Box: 77843, College Station, TX, USA; E-mails: srsurani@hotmail.com, Surani@tamu.edu

Published: August 23, 2024

*Cite as:* El Labban M, El-Zibaoui R, Usama S, Niaz F, Cohen A, Krastev P, Khan S, Surani S. Malnutrition and Obesity in Patients with COPD Exacerbation, Insights from the National Inpatient Sample. Open Respir Med J, 2024; 18: e18743064322829. http://dx.doi.org/10.2174/0118743064322829240801094830

***Send Orders for Reprints to reprints@benthamscience.net***

**Supplemental Table 1. Diagnosis and corresponding ICD-10 codes.**

| Diagnosis | ICD-10 |
|---|---|
| Malnutrition | E43, E440, E441, E46 |
| Sepsis | H594,T802,T826,T827,T835,T836,T845,T847,T857,O860,R5082, K6811,T880,T802,R7881,A400,A401,A403,A408,A409,A427,A427, B377,A267,A282,A5486,B007,A327,A241,A392,A394,A207,A217,A483, R578,A419,A4101,A4102,A411,A412,A414,A4150,A4151,A4152,A4153, A4159,A4181,A4189,A419 |
| Diabetes Mellitus Type II | E1100,E1101,E1111,E1122, E1129, E1131,E11311,E11319,E11321,E113211, E113212,E113213, E113219,E113291,E113292,E113293,E113299,E113311,E113312, E113313,E113319,E113391,E113392,E113393,E113399,E113411,E113412, E113413, E113419,E113491,E113492,E113493,E113499,E113511,E113512,E113513,E113519, E113521,E113522,E113523,E113529,E113531,E113532,E113533,E113539,E113541, E113542,E113543,E113549,E113551,E113552,E113553,E113559,E113591,E113592, E113593,E113599,E1136,E1137X1,E1137X2,E1137X3,E1137X9, E1139,E1140, E1141,E1142,E1143, E1144,E1149,E1151,E1152,E1159,E1161,E11610,E11618, E11620,E11621, E1162,E11628,E11630, E11638,E11641,E11649, E1165,E1169, E118,E119 |
| Essential Hypertension | I110,I119,120, I129, I1310,I1311,I132, I130, I150, I151, I152, I158, I159, I160, I161, I169 |
| Chronic kidney disease | N181, N182, N1830, N1831, N1832, N184, N185, N18, N189 |

| Diagnosis | ICD-10 |
|---|---|
| Dementia | F0150, F01511, F01518, F0152, F0153, F0154, F01A0, F01A11, F01A18, F01A2, F01A3, F01A4, F01B0, F01B11, F01B18, F01B2, F01B3, F01B4, F01C0, F01C11, F01C18, F01C2, F01C3, F01C4, G300, G301, G308, G309, G3183, F0390, F03911, F03918, F0392, F0393, F0394, F03A0, F03A11, F03A18, F03A2, F03A3, F03A4, F03B0, F03B11, F03B18, F03B2, F03B3, F03B4, F03C0, F03C11, F03C18, F03C2, F03C3, F03C4 |
| Pulmonary Hypertension | I270, I2720 I 2721 I2722 I2723 I2724 I2729 |
| Chronic obstructive pulmonary disease exacerbation | J440 J441 |
| Coronary Artery Disease | I4811, I2510, I252, I25810, I25811, I25812, I2582, I2583, I2584, I2589, I259, I200, I201, I202I, I208, I209, I25110, I25111, I25112, I25118, I25119 |
| Chronic heart failure | I5032, I5042 |
| Sarcopenia | M6284 |
